# Honeybee product therapeutic as stem cells homing for ovary failure

**DOI:** 10.14202/vetworld.2016.1324-1330

**Published:** 2016-11-29

**Authors:** Erma Safitri, Thomas V. Widiyatno, R. Heru Prasetyo

**Affiliations:** 1 Department of Veterinary Reproduction, Faculty of Veterinary Medicine, Universitas Airlangga, Surabaya, East Java, Indonesia; 2 Stem Cells Research Division of Institute Tropical Disease, Universitas Airlangga, Surabaya, East Java, Indonesia; 3 Department of Veterinary Pathology, Faculty of Veterinary Medicine, Universitas Airlangga, Surabaya, East Java, Indonesia; 4 Department of Parasitology, Faculty of Medicine, Universitas Airlangga, Surabaya, East Java, Indonesia

**Keywords:** honeybee product, ovary failure, stem cells homing

## Abstract

**Aim:**

Complexity of the method of isolation, cultivation *in vitro* and the expensive cost of transplantation process of stem cells, it would require an innovation to homing and differentiation of stem cells and increase folliculogenesis. The stem cells homing was achieved through the provision of food or beverages derived from natural materials like honeybee product. Through honeybee product, there will be homing of stem cells and accompany with the sources from the body itself will take place in regeneration of the ovary.

**Materials and Methods:**

Female rats model of degenerative ovary was obtained through food fasting but still have drinking water for 5 days. It caused malnutrition and damage of the ovarian tissue. The administration of 50% honeybee product (T1) was performed for 10 consecutive days, while the positive control group (T0+) was fasted and not given honeybee product and the negative control (T0−) not fasted and without honeybee product. Observations were taken for homing of stem cells, raised of folliculogenesis, differentiation of stem cells, and regeneration of the ovarian tissue using routine H&E staining.

**Results:**

Homing of stem cells shown the vascular endothelial growth factor and granulocyte colony-stimulating factor expression; enhancement of folliculogenesis was indicated by an increase of follicle dee Graaf count; enhancement of differentiation of stem cells was indicated by growth differentiation factor-9 expression; and regeneration of ovarian tissue indicated by intact ovarian tissue with growing follicles.

**Conclusion:**

Honeybee product can be induced endogenous stem cells in regeneration of ovary failure due to malnutrition.

## Introduction

The interest of stem cell therapy today and the next few decades tends to be greatly increased [1-3]. The stem cells have tremendous promise to treat a range of diseases. Stem cell transplantation provides new hope in the treatment of various diseases including infertility due to degenerative conditions of the ovary which could not be cured through usual treatment and operative measures [3-7].

However, because of the complexity of the method of isolation [8], cultivation *in vitro* [9] and the expensive cost of transplantation process of stem cells [10], it would require an innovation to homing of stem cells and increase immune response [4,11,12] and in the same time induced a differentiation of endogenous stem cells [11,13], and therefore, avoid the expensive process of transplantation. Automobilization and increased immune response accompanied with the differentiation of stem cells was achieved through the provision of food or beverages derived from natural materials like honeybee product [3,4,11,14].

The recent study performed a provision of honeybee product [3,4,11,15,16]. Through the administration of honeybee products, it was expected a homing and differentiation of stem cells the patients with ovary failure [4,17]. The presence of homing and differentiation of stem cells is made from the body itself and it will regenerate the follicles of the ovary.

The regeneration of ovary can be proven microscopically and also on molecular level [13,18]. The histological appearance will reveal regeneration of ovarian tissue at molecular level there were evident of several expressions such as CD34+ and CD45+ of hematopoietic stem cells (HSCs) [4,19], expression of transforming growth factor-ß (TGF-ß) [11], vascular endothelial growth factor (VEGF), granulocyte colony-stimulating factor (G-CSF) [11,20,21], and growth differentiation factor-9 (GDF-9) of the ovary [20,21].

The aim of the study was using honeybee product therapeutic will be homing of stem cells (based on VEGF and G-CSF expression), increase of folliculogenesis count and stem cells differentiation (based on GDF-9 expression) and accompany with the sources from the body itself will take place in regeneration of the ovary (based on histopathological observation with H&E staining) on rat model with ovary failure due to malnutrition.

## Materials and Methods

### Ethical approval

The present study was approved by ethical committee vide Ethical Clearance No: 064-KE (Komisi Etik Penelitian, Fakultas Kedokteran Hewan Universitas Airlangga, Animal Care and Use Committee (ACUC)).

### Ovarian degeneration modeling

Malnutrition which causes the ovaries degeneration in female rats was performed following food fasting for 5 consecutive days, but they still have water to drink *ad libitum* using feeding tube [22]. The laboratory animals used in this study were healthy female Wistar rats, 12-14 week-old and each 250-300 g weight [23]. Healthy condition was determined by their active movement. Rats kept in an individual plastic cage in laboratory for Experimental Animal of Veterinary Medicine, Faculty of Universitas Airlangga with adequate ventilation.

Treatment: The study was divided into three groups, each has 15 replications. They were:

1. The negative control group (T0−): Rats with ovary normal (not fasted) and without honeybee product
2. The positive control group (T0+): Rats with ovary failure (fasted for 5 days) and without honeybee product
3. The treatment Group 2 (T1): Rats ovary failure (fasted for 5 days), given 50% honeybee product in the drinking water for the next 10 days after fasted.

Honeybee products that used in this study were raw honeybee products from Batu Malang East Java, Indonesia. Observations were taken for homing stem cells, raised of folliculogenesis, differentiation of stem cells, and regeneration of the ovarian tissue. Homing of stem cells was shown by VEGF and G-CSF expressions [11,21]. Raised of folliculogenesis was indicated by an increase of follicle dee Graaf expression [24]. Differentiation of stem cells into progenitor cells presented by the expression of GDF-9 using immunohistochemistry technique in ovarian tissue [20,21] and regeneration of the ovarian tissue using routine HE staining [25].

### Immunohistochemical (IHC) methods for observation of VEGF, G-CSF, and GDF-9

IHC observation was performed to determine the expressions of VEGF, G-CSF, and GDF-9 [11,21,25]. First, made an incision through ovarian tissues transversely from paraffin blocks [25]. The IHC techniques using monoclonal antibodies anti-VEGF, anti-G-CSF, and anti-GDF-9. Observations of VEGF, G-CSF, and GDF-9 expressions were made using a light microscope with a magnification of 200 times. The expression of each variable is indicated by the number of cells with brownish discoloration due to DAB-chromogen in each incision [25,26].

### Histological and follicle dee Graaf observation of ovary

Identification of follicle dee Graaf and regenerate ovarian tissues performed through light microscopy examination [27]. Histological preparations such as the following: Fixation of rat ovary in 10% buffer formalin. Subsequently dehydration with a series of alcohol, i.e., from 70%, 80%, 90%, and 96% (absolute). Clearing of the ovary of rat in xylene solution. The tissues were infiltrated with embedding agent, the liquid paraffin. The sectioning was done with microtome that could be set with a distance at 4-6 µ, and the sections were placed on a slide. The embedding process must be reversed to get the paraffin wax out of the tissue and allow water soluble dyes to penetrate the sections. Therefore, before any staining can be done, the slides are “deparaffinized” by running them through xylenes to alcohols to water. The staining used was the routine H & E. The stained section then mounted with Canada balsam and placed a coverslip on it [25].

Observations and identifications of follicle dee Graaf and ovarian regenerations are based on the histological measures of that of the normal tissue [4,25].

### Statistical analysis

Expressions of follicle dee Graaf, VEGF, G-CSF, and GDF-9 were statistically analyzed using SPSS 15 for Windows XP with the level of significance 0.05 (p=0.05) and the confidence level 99% (α=0.01). Steps of comparative hypothesis tests are as follows: Test data normality with the Kolmogorof–Smirnov test, homogeneity of variance test, analysis of variance factorial, and *post-hoc* test (least significant difference test) using the Tukey HSD 5%.

## Results and Discussion

The collected data from 45 female rats were divided into three groups: Negative control group (T0−) is normal ovary without honeybee product; positive control group (T0+) is ovary failure without honeybee product; (T1) group is ovary failure + 50% honeybee product in drinking water for 10 days. In detail, the results of the study are as follows: The effectiveness of honeybee product was based on: (1) Homing of stem cells based on VEGF and G-CSF expressions, (2) follicle dee Graaf count, (3) GDF-9 expressions, and (4) regeneration of ovarian tissue.

**Figure-1:**
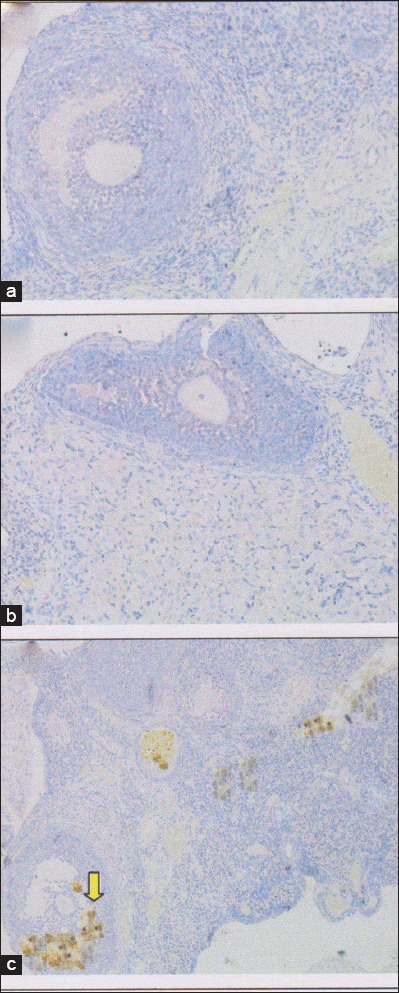
Homing of stem cells based on vascular endothelial growth factor (VEGF) expression in rat ovarian tissue by immunohistochemical. (a) Control negative group (T0−), with normal ovary without honeybee product: Score of VEGF expression=0.45±0.85^a^; (b) control positive group (T0+), with ovary failure without honeybee product: Score of VEGF expression=0.25±0.65^a^, (c) T1 group, the ovary failure +50% honeybee product in drinking water for 10 days: Score of VEGF expression 2.75±0.55^b^. The different superscripts indicate significant difference at p<0.05.

**Figure-2:**
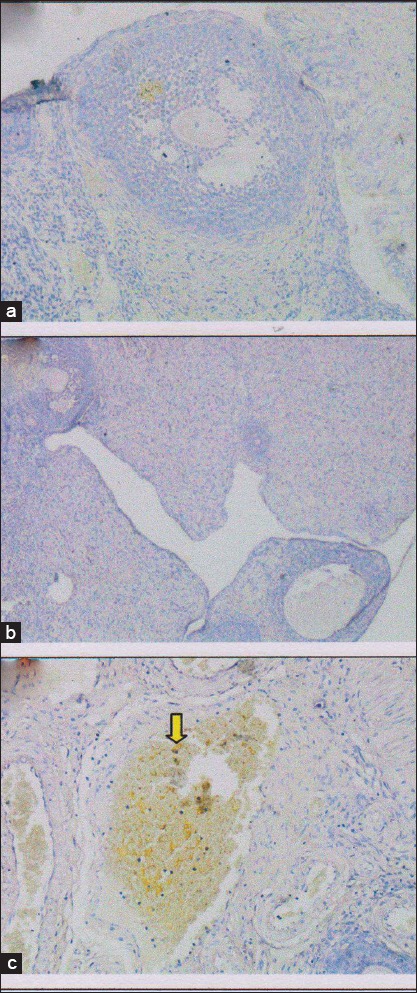
Homing of stem cells based on granulocyte colony-stimulating factor (G-CSF) expression in rat ovarian tissue by immunohistochemical. (a) Control negative group (T0−), with normal ovary without honeybee product: Score of G-CSF expression=0.75±0.35^a^, (b) control positive group (T0+), with ovary failure without honeybee product: Score of G-CSF expression=0±0^a^, (c) T1 group, the ovary failure +50% honeybee product in drinking water for 10 days: Score of G-CSF expression 2.95±0.43^b^. The different superscripts indicate significant difference at p<0.05.

Homing of stem cells analyzed by IHC based on increased of VEGF and G-CSF expression. The analysis showed that: Nonsignificant of the negative control group (T0−) and the positive control group (T0+) showed homing of stem cells, based on the lower expression of VEGF and G-CSF with average were <1 (Figures-1 and 2), while the (T1) group has shown homing of stem cells based on the average of VEGF and G-CSF=2.75±0.55^b^ and 2.95±0.43^b^ (Figures-1 and 2). Based on statistical analysis T1 group was significantly different (p<0.05) than the other two treatments (T0− and T0+), but among the latter two treatments no significant difference (p>0.05) (Table-1).

**Table 1:** Analysis of average VEGF, G-CSF and GDF-9 with immunohystochemical staining and Follicle dee Graaf count.

Treatments	Score±SD			Average follicle dee Graaf Count±SD

	Average VEGF expression	Average G-CSF expression	Average GDF-9 expression	
Negative control group (T0−) with normal ovary without honeybee product	0.45±0.85^a^	0.75±0.35^a^	2.95±0.41^c^	7±0.85^c^
Positive control group (T0+) with ovary failure without honeybee product	0.25±0.65^a^	0±0^a^	0±0^a^	0.75±0.125^a^
T1 group, the ovary failure+50% honeybee product in drinking water for 10 days	2.75±0.55^b^	2.95±0.43^b^	2±0.45^b^	5.416±0.807^b^

a,b,c Values in the same column with different superscripts indicate significant difference at p<0.05 (n=15). VEGF=Vascular endothelial growth factor, G-CSF=Granulocyte colony-stimulating factor, GDF-9=Growth differentiation factor-9

Furthermore, enhancement of folliculogenesis based on follicle dee Graaf expression, in the normal control group (T0−) where its count was 7±0.845^c^. The group was significantly different (p<0,05) than the group of ovary failure (T0+) was 0.75±−0.125^a^ and The group with ovary failure +50% honeybee product (T1) was 5.416±0.807^b^ (Table-1). However, the group of ovary failure (T0+) has the lowest follicle dee Grasf expression. The group of ovary failure (T0+) was significantly different (p<0,05) than the group with ovary failure +50% honeybee product (T1).

**Figure-3:**
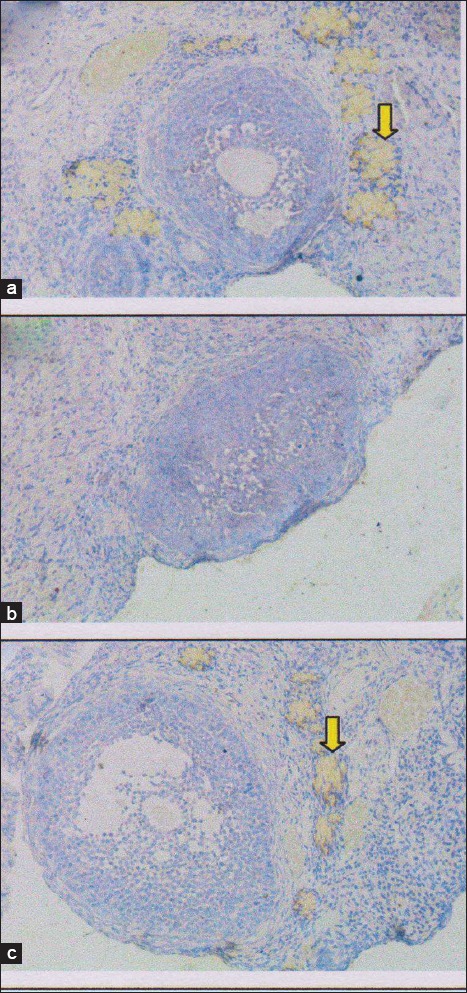
Growth differentiation factor-9 (GDF-9) expression in rat ovarian tissue shown by immunohistochemical staining on several treatments. (a) Control negative group (T0−), with normal ovary without honeybee product: Score of GDF-9 expression=2.95±0.41^c^, (b) control positive group (T0+), with ovary failure without honeybee product: Score of GDF-9 expression=0±0^a^, (c) T1 group, the ovary failure +50% honeybee product in drinking water for 10 days: Score of GDF-9 expression 2±0.45^b^. The different superscripts indicate significant difference at p<0.05.

The effectiveness of honeybee product is based on GDF-9 expression as a result of the differentiation of the progenitor cells. Expression of GDF-9 in the group with 50% honeybee product (T1) had scored 2±0.43^b^ (GDF-9 expression between 25% and 50%). Although, the T1 group’s score was lower than the normal control group (T0−) with the score was 2.95±0.41^c^ and GDF-9 expression >50%, but still higher than the (T0+) group with ovary failure, which was not expressed at all (0±0^a^ and GDF-9 expression 0%) (Table-1 and Figure-3).

**Figure-4:**
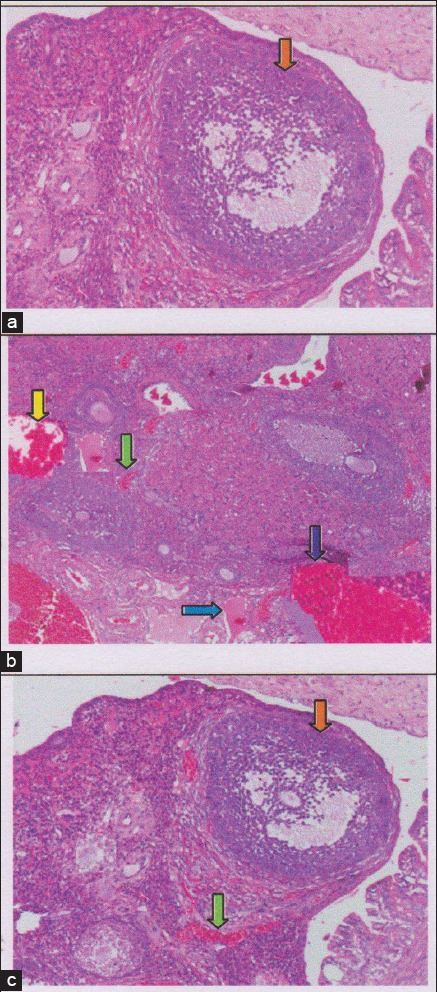
The regeneration of ovarian tissue indicated by microscopic examination with hematoxylin and eosin (HE) staining in rat ovarian tissue in a few treatments. (a) Control negative group (T0−), with normal ovary without honeybee product was shown growing follicles (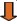); (b) the group of ovary failure (T0+), congestion of ovarian (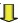), and severe hemorrhage (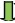), also visible hemosiderin (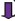) due to blood cell lysis (brownish yellow color) with deposition of fibrin (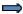) indicating that chronic congestive has been occurred, (c) the group ovary failure + 50% honeybee product (T1), ovaries begin to regenerate so it looks intact, although there is still a slight hemorrhage and congestion in some areas, but has seen growing follicles (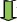).

In this study, the regeneration of the ovary could be observed histologically with H & E staining. Microscopic examination showed that the group with ovary failure +50% honeybee product (T1) did the best ovarian tissue repair. The tissue repair was determined by the regeneration of ovary which its follicles grew normally. The appearance of this improvement could be compared with the negative control group (T0−) which did not undergo ovarian failure and remains in normal condition and the follicles grew normally. The appearance of the tissue damage could be compared with the positive control rat (T0+) which experiencing ovarian degeneration. The group with ovary failure (T0+), was congested and showed severe hemorrhage, formation of hemosiderin granules due to erythrolysis (brownishyellow color) accompanied with fibrosis indicating that chronic inflammation has been occurred [28] (Figure-4).

This study showed that administration of 50% honey for 10 days (T1) can be used for the treatment of female rat with ovary failure model caused malnutrition. The effectiveness of honeybee product was based on: (1) Homing of stem cells (based on VEGF and G-CSF expressions); (2) follicle dee Graaf count; (3) GDF-9 expressions; (4) regeneration of ovarian tissue.

Homing of stem cells (based on VEGF and G-CSF expressions) using IHC method [11,21]. Increase of follicle dee Graaf count was indicated of folliculogenesis raised [27]. Differentiation of stem cells into progenitor cells presented by the expression of GDF-9 using IHC technique in ovarian tissue [20,21,29]. Regeneration of ovarian tissue observation with routine HE staining [4].

Homing of stem cells could be performed by inducing the stem cells to mobilize toward the defect area [13,30]. Mobilization of stem cells toward defect area for engraft in tissue, and then, the cells have a function and repair effect. Furthermore, the cells have function including secretion of soluble mediator that occurred cooperation between the host cells with mobilization cells from exogenous stem cells that have paracrine effect [21]. The process of homing can occur in several ways, one of which is enhancement of the immune response that induced by an inflammatory reaction due to injurious signals (cytokines, nuclear factor κB, Wnt through β catenin) from the damaged tissue [25]. In this study, injury due to malnutrition signal causes an increase in cytokines so that the alteration of ovarian tissue as the primary network of the female reproductive system could be inevitable [26]. Some cytokines that induction of stem cells for the migration and homing in the area of injury, in this study, is the VEGF and G-CSF [21].

Homing mechanism starts from HSCs out of the bone marrow through chemokine receptor (CCXR4) to locate the homing signal factor of stromal-derived factor 1 (SDF1) [4]. Furthermore, osteoblasts (cell progenitors) in cell adhesion proteins (cell adhesion proteins), among others VICAM. Furthermore, G-CSF stimulates an increase in neutrophils in the bone marrow and then increase the blood cells. Neutrophils increases will produce protease enzymes including elastase protein damage for stem cell homing signal such as SDF1 and VICAM and then caused the stem cells out of the bone marrow. After that, quiescent stem cells out into the blood flow. The role of honey administered orally in this research was caused an increase of G-CSF [21].

Furthermore, the ovarian tissue repair based on GDF-9 expression (Table-1). GDF-9 which is progenitor cells of germline stem cells will stimulate ovarian cortex cells proliferation [31]. The T1 group (ovary failure +50% honeybee product) score of GDF-9 expression was 2±0.43^b^. Although the score is below the negative control group (T0−) was significantly different (p<0,05), with the score of which 2.95±0.41^c^, but the score is still above the group of ovary failure without honeybee products (T0+) which shown no expression at all (0±0^a^) (Table-1 and Figure-3). This is in accordance with the opinion that honeybee product causes the stem cells develop rapidly and differentiate into cells that are needed as a response to injury and enhance the immune response itself [32].

GDF-9 is the growth factor derived from TGF-ß family was produced by the oocyte. GDF-9 is a crucial factor in folliculogenesis and fertility [33]. Furthermore, progenitor germ cells will repair the damaged follicles and also can repair molecular communication in ovarian follicles by increasing the production of SCF and GDF-9, so as to overcome the problems of folliculogenesis due to malnutrition [21]. Homing will be activated oogenesis directly or indirectly. Directly through activation of cells inhibited and will indirectly stimulate the microenvironment (niche) of damaged cells [34].

Regeneration of ovarian tissue shown as an intact ovarian tissue with growing follicles is the third and fourth identification of the effectiveness from the use of honeybee product. In this study, the regeneration of the ovarium can be observed microscopically with HE staining [31,35]. Microscopic examination showed that 50% honeybee product administration (T1), leading to the ovarian tissue repair. Improvements are identified based on the regeneration of the ovary with growing follicles. Overview of these improvements can be compared with a negative control group (T0−) which did not suffer from ovarian failure and remains in normal condition with growing follicles (Figure-4). The abnormal feature of the damaged ovary can be compared with positive control group of rat (T0+) rat with ovarian degenerative. The latter shown congested and there were severe hemorrhage and hemosiderosis (yellow-brown color) due to the hemolysis of the red blood cells with fibrin deposition, indicating that chronic congestion has been occurred (Figure-4).

## Conclusions

Treatment of 50% honeybee product for 10 days in female rat with ovary failure reveals: (1) Homing of stem cells, shown the VEGF and G-CSF markers which are expressed in ovarian tissue with IHC; (2) enhancement of folliculogenesis was indicated by increase of follicle dee Graaf count; (3) enhancement of GDF-9 expression in ovarian tissue with IHC staining; (4) regeneration of ovarian tissue indicated by intact ovarian tissue with growing follicles; although there is still slight hemorrhage and congestion, hemosiderin granules and fibrin deposition do not exist.

## Authors’ Contributions

ES: Research coordinator and flowcytometri method, drafted and revised the manuscript. TVW: Method of Immunohistochemical, Histophatology Anatomy and statistical analysis. RHP: Prepared female rats model of degenerative ovary due to malnutrition and administration of 50% honeybee product. All authors read and approved the final manuscript.
